# Correction: Low level of plasma DNase is associated with worse clinical outcome in testicular germ cell tumor patients and exogeneous DNase I improves cisplatin treatment efficacy

**DOI:** 10.1371/journal.pone.0340033

**Published:** 2025-12-31

**Authors:** Michal Mego, Barbora Vlkova, Katarina Kalavska, Michal Pastorek, Zuzana Cierna, Zuzana Sestakova, Miroslav Chovanec, Natalia Udvorkova, Lucia Kucerova, Peter Celec

The word “(Leverages)” should not be included in the article title. The correct title is: Low level of plasma DNase is associated with worse clinical outcome in testicular germ cell tumor patients and exogeneous DNase I improves cisplatin treatment efficacy.

Additionally, the author initials appear incorrectly in the citation. The correct citation is: Mego M, Vlkova B, Kalavska K, Pastorek M, Cierna Z, Sestakova Z, et al. (2025) Low level of plasma DNase is associated with worse clinical outcome in testicular germ cell tumor patients and exogeneous DNase I improves cisplatin treatment efficacy. PLoS One 20(12): e0336190. https://doi.org/10.1371/journal.pone.0336190.
