# Correction: Low level of plasma DNase is associated with worse clinical outcome in testicular germ cell tumor patients and exogeneous DNase I improves cisplatin treatment efficacy

**DOI:** 10.1371/journal.pone.0344953

**Published:** 2026-03-16

**Authors:** The PLOS One Staff

## Notice of Republication

This article was republished on February 23, 2026, to correct errors in the title and citation that were introduced during the typesetting process. The correct citation is: Mego M, Vlkova B, Kalavska K, Pastorek M, Cierna Z, Sestakova Z, et al. (2025) Low level of plasma DNase is associated with worse clinical outcome in testicular germ cell tumor patients and exogeneous DNase I improves cisplatin treatment efficacy. PLoS One 20(12): e0336190. https://doi.org/10.1371/journal.pone.0336190.

The publisher apologizes for the errors. Please download this article again to view the correct version. The originally published, uncorrected article and the republished, corrected articles are provided here for reference.

## Supporting information

S1 File Originally published, uncorrected article. (PDF)

S2 File Republished, corrected article. (PDF)

## References

[1] Mego M, Vlkova B, Kalavska K, Pastorek M, Cierna Z, Sestakova Z, et al. Low level of plasma DNase is associated with worse clinical outcome in testicular germ cell tumor patients and exogeneous DNase I improves cisplatin treatment efficacy. PLoS One. 2025;20(12):e0336190. doi: 10.1371/journal.pone.0336190 41343562 PMC12677466

