# Supplementary figures and images for: Low level of plasma DNase is associated with worse clinical outcome in testicular germ cell tumor patients and exogeneous DNase I improves cisplatin treatment efficacy
Source: PLoS One. 2025 Dec 4;20(12):e0336190. doi: 10.1371/journal.pone.0336190 (PMC12677466; doi:10.1371/journal.pone.0336190)

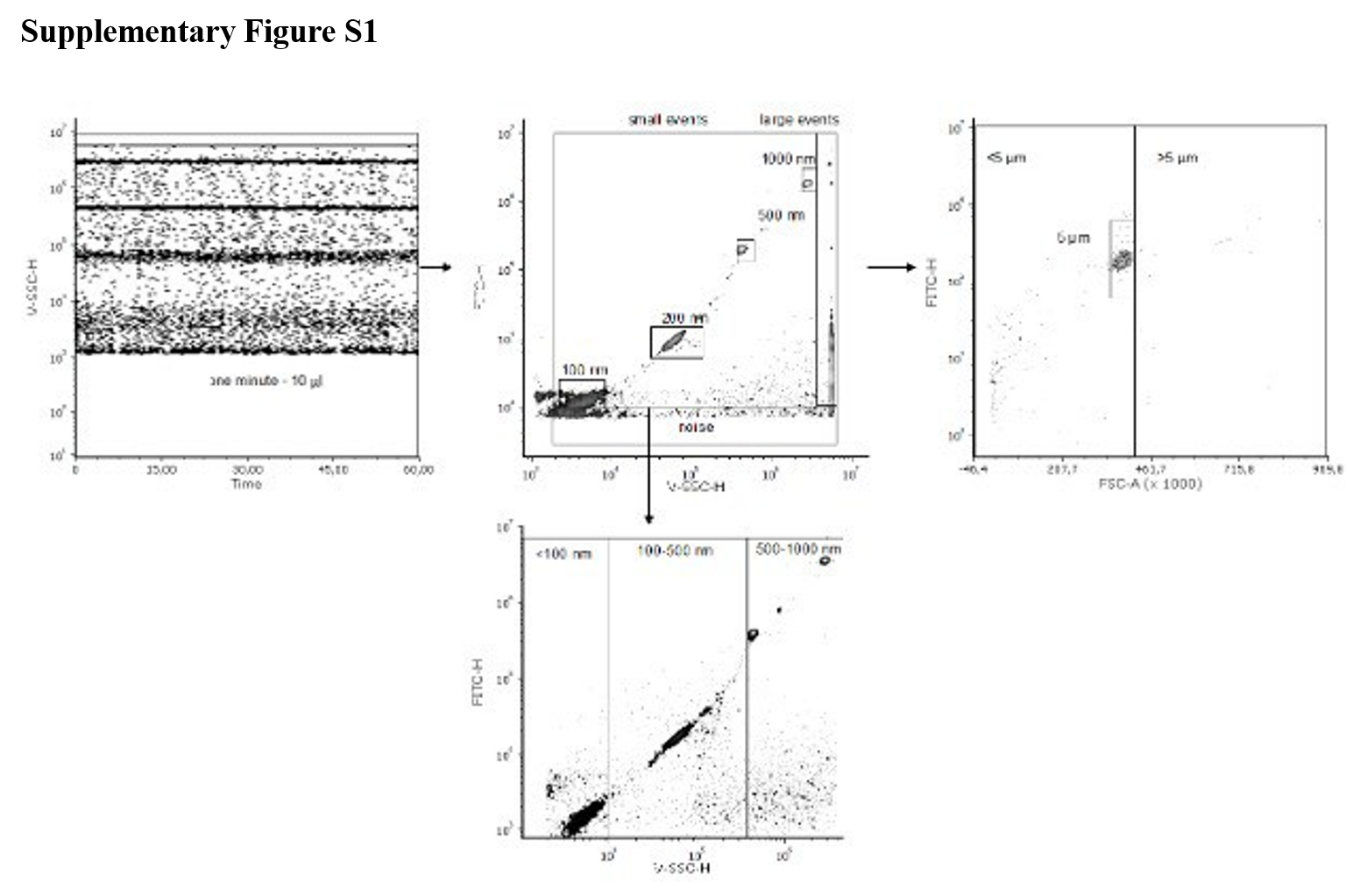

Supplement: S1 Fig — All events were gated according to time for one minute which corresponds to 10 µL of sample volume. Calibration beads and blank sample were then used to create specific size gates and set the threshold on FITC-H channel for noise. Particles divided into small and large events were then separated according to size, as shown above. (TIF) [file pone.0336190.s001.tif]

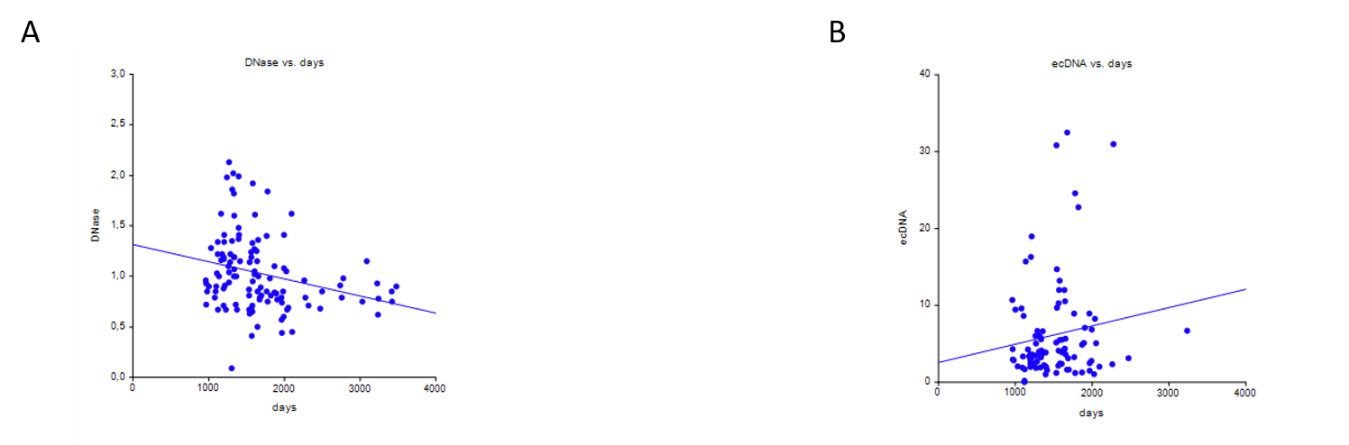

Supplement: S2 Fig — (B) ecDNA vs. days of samples storage: Spearmen correlation = 0.0874, p = 0.3996. (TIF) [file pone.0336190.s002.tif]

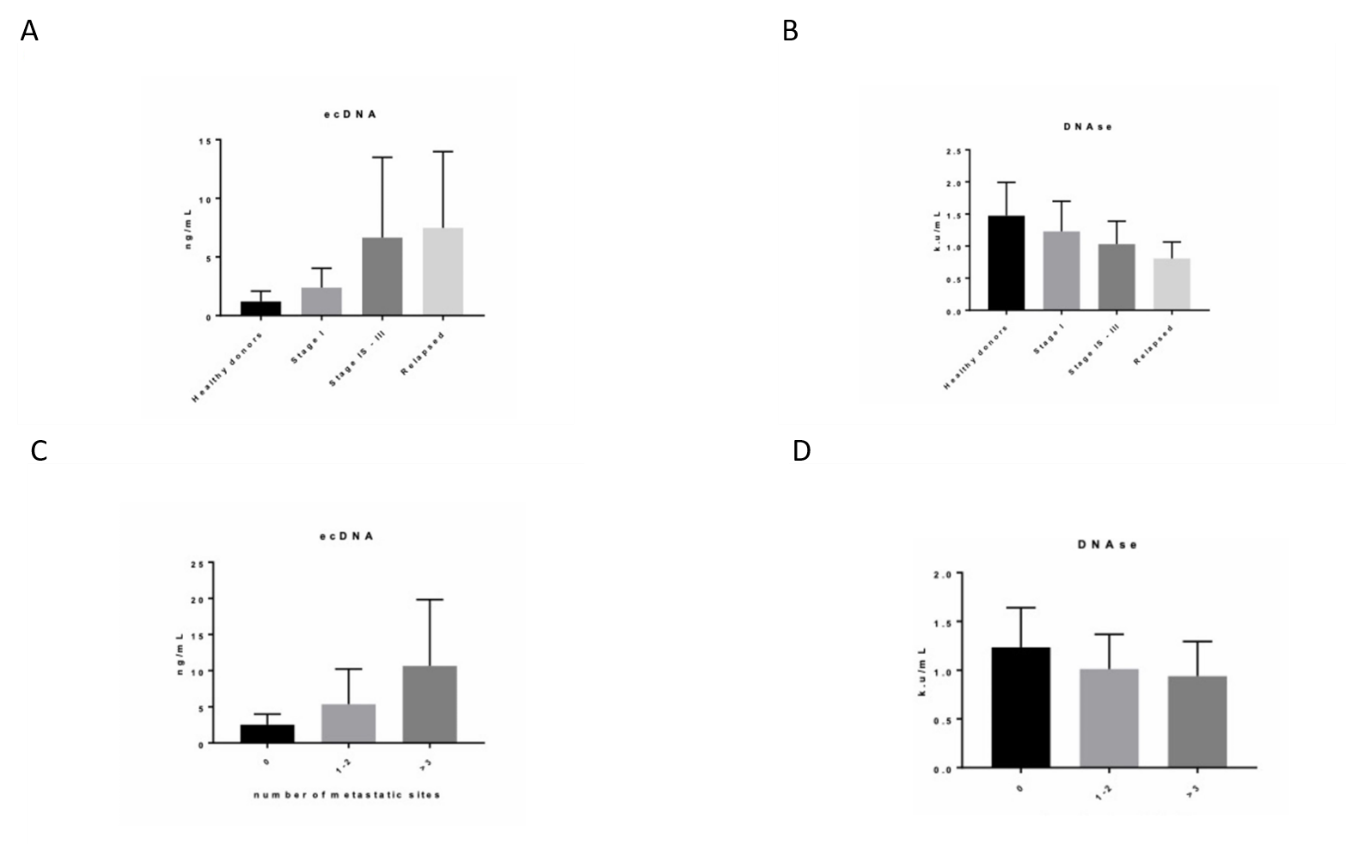

Supplement: S3 Fig — (TIF) [file pone.0336190.s003.tif]

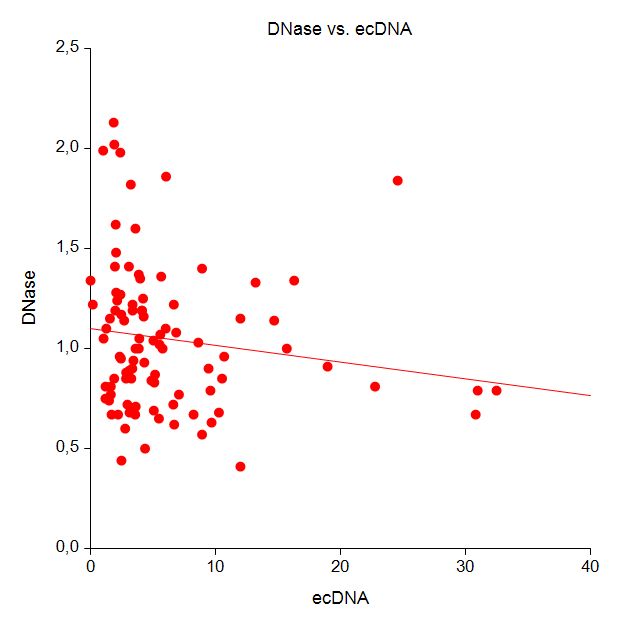

Supplement: S4 Fig — (TIF) [file pone.0336190.s004.tif]

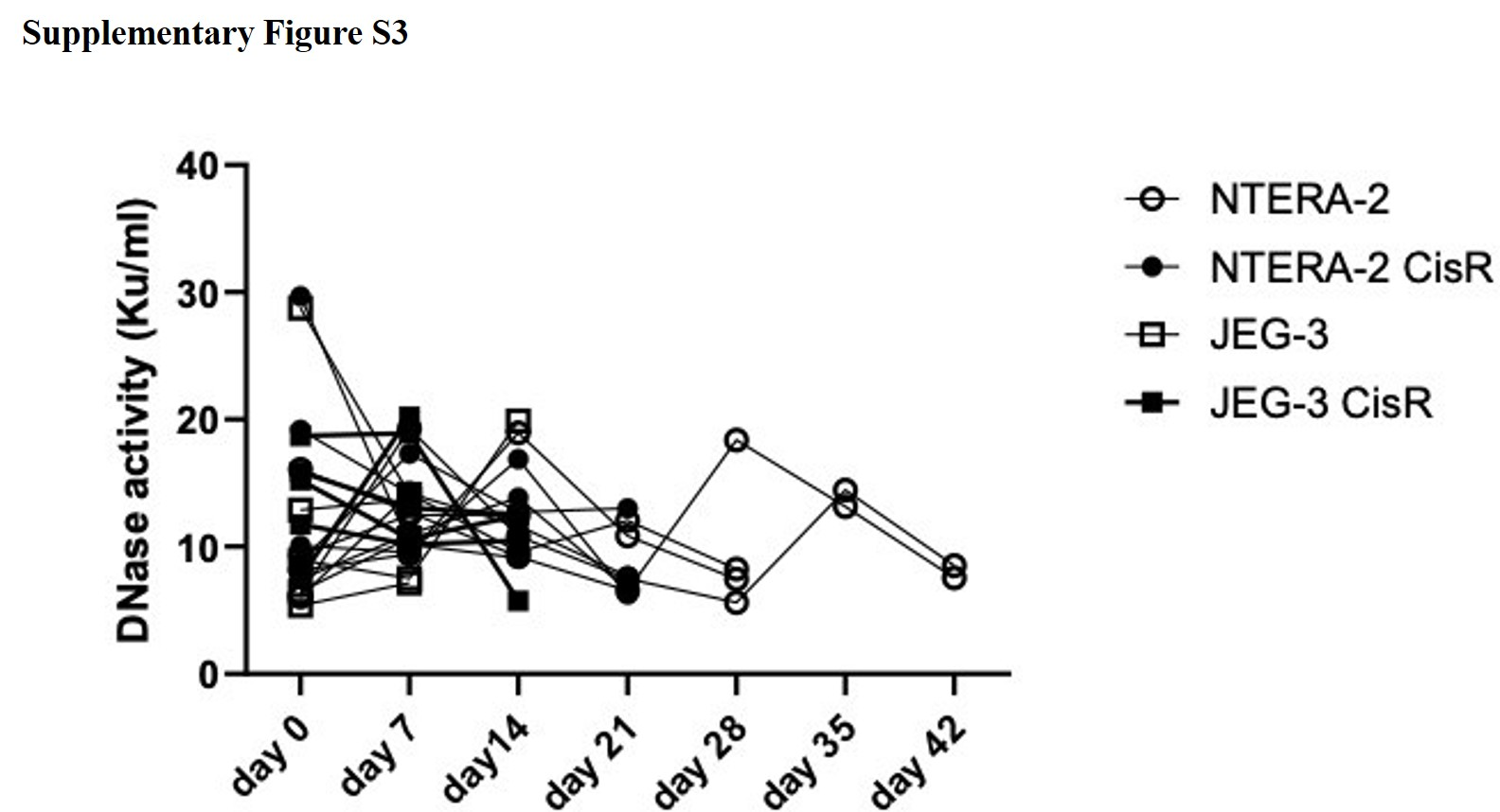

Supplement: S5 Fig — (TIF) [file pone.0336190.s005.tif]
