# Supplementary material for: Low level of plasma DNase is associated with worse clinical outcome in testicular germ cell tumor patients and exogeneous DNase I improves cisplatin treatment efficacy
Source: PLoS One. 2025 Dec 4;20(12):e0336190. doi: 10.1371/journal.pone.0336190 (PMC12677466; doi:10.1371/journal.pone.0336190)
Supplement: S1 Table — (DOCX) [file pone.0336190.s006.docx]

**Supplementary Table 1.** Comparison of ecDNA, DNase and markers of NETosis in GCTs patients and HDs.

| **Variable** | **N** | **Mean** | **Median** | **SD** | **SEM** | ***p* value** |
| --- | --- | --- | --- | --- | --- | --- |
|  |  |  |  |  |  |  |
| **Plasma total ecDNA ng/mL** |  |  |  |  |  |  |
| TGCT patients | 95 | 6.2 | 3.9 | 6.5 | 0.6 | **0.00015** |
| HDs | 8 | 1.2 | 1.1 | 0.9 | 2.2 |  |
| **Plasma ncDNA GE/mL** |  |  |  |  |  |  |
| TGCT patients | 88 | 6683.8 | 2996.0 | 10167.5 | 1009.6 | **0.02868** |
| HDs | 15 | 2515.1 | 2232.0 | 2156.3 | 2445.3 |  |
| **Plasma mtDNA GE/mL** |  |  |  |  |  |  |
| TGCT patients | 97 | 169606.6 | 101693.0 | 199711.3 | 18724.2 | 0.11289 |
| HDs | 19 | 85315.9 | 76536.0 | 51622.3 | 42307.0 |  |
| **Plasma DNase K.U./mL** |  |  |  |  |  |  |
| TGCT patients | 117 | 1.0 | 1.0 | 0.4 | 0.0 | **0.00049** |
| HDs | 19 | 1.5 | 1.5 | 0.5 | 0.1 |  |
| **Pellet total ecDNA ng/mL** |  |  |  |  |  |  |
| TGCT patients | 91 | 2.7 | 1.9 | 3.3 | 0.3 | **0.03441** |
| HDs | 19 | 1.9 | 1.3 | 2.2 | 0.7 |  |
| **Pellet ncDNA GE/mL dich** |  |  |  |  |  |  |
| TGCT patients | 75 | 80911.0 | 4164.0 | 276972.1 | 29692.7 | 0.47487 |
| HDs | 15 | 43675.0 | 4164.0 | 100762.0 | 66395.0 |  |
| **Pellet mtDNA GE/mL** |  |  |  |  |  |  |
| TGCT patients | 98 | 411882.5 | 83753.0 | 973588.4 | 90741.2 | **0.00001** |
| HDs | 18 | 27943.2 | 9906.5 | 52057.6 | 211729.5 |  |
| **< 100 nm** |  |  |  |  |  |  |
| TGCT patients | 98 | 107977.6 | 49500.0 | 156174.2 | 15298.1 | 0.21798 |
| HDs | 8 | 52450.0 | 31900.0 | 52629.6 | 53543.4 |  |
| **100-500 nm** |  |  |  |  |  |  |
| TGCT patients | 98 | 696097.9 | 442000.0 | 887336.1 | 87225.7 | 0.54983 |
| HDs | 8 | 617950.0 | 503500.0 | 408755.3 | 305289.9 |  |
| **500-1000 nm** |  |  |  |  |  |  |
| TGCT patients | 98 | 955781.6 | 854400.0 | 611798.4 | 59884.1 | 0.28174 |
| HDs | 8 | 1016150.0 | 985000.0 | 186229.5 | 209594.4 |  |
| **< 5** μ**M** |  |  |  |  |  |  |
| TGCT patients | 98 | 1005894.0 | 275000.0 | 1381706.0 | 134806.2 | 0.11167 |
| HDs | 8 | 194750.0 | 199900.0 | 68119.5 | 471821.8 |  |
| **> 5** μ**M** |  |  |  |  |  |  |
| TGCT patients | 98 | 427977.6 | 131200.0 | 702157.1 | 68567.1 | 0.73771 |
| HDs | 8 | 156625.0 | 126300.0 | 115749.2 | 239984.9 |  |
| **Small particles (< 1** μ**M)** |  |  |  |  |  |  |
| TGCT patients | 98 | 1759857.0 | 1447100.0 | 1385358.0 | 135950.1 | 0.48413 |
| HDs | 8 | 1686550.0 | 1540500.0 | 561741.4 | 475825.3 |  |
| **Large particles (> 1** μ**M)** |  |  |  |  |  |  |
| TGCT patients | 98 | 1433871.0 | 424800.0 | 1936052.0 | 188930.3 | 0.19646 |
| HDs | 8 | 351375.0 | 319800.0 | 175239.3 | 661256.0 |  |
| **All particles** |  |  |  |  |  |  |
| TGCT patients | 98 | 3193729.0 | 2179500.0 | 2618657.0 | 255883.1 | 0.52615 |
| HDs | 8 | 2037925.0 | 1912400.0 | 556671.3 | 895591.0 |  |
| **MPO (ng/mL)** |  |  |  |  |  |  |
| TGCT patients | 99 | 10.3 | 6.9 | 9.1 | 0.8 | **0.00175** |

| HDs | 19 | 5.0 | 4.4 | 2.5 | 1.9 |  |
| --- | --- | --- | --- | --- | --- | --- |
| **NE (ng/mL)** |  |  |  |  |  |  |
| TGCT patients | 76 | 3.0 | 2.4 | 2.2 | 0.2 | **0.00225** |
| HDs | 13 | 1.6 | 1.2 | 1.2 | 0.6 |  |

**Abbreviations:** ecDNA, extracellular DNA, ncDNA, nuclear DNA, mtDNA, mitochondrial DNA , MPO, myeloperoxidase, NE, neutrophil elastase, SD, standard deviation, SEM, standard error of mean
