# Supplementary material for: Low level of plasma DNase is associated with worse clinical outcome in testicular germ cell tumor patients and exogeneous DNase I improves cisplatin treatment efficacy
Source: PLoS One. 2025 Dec 4;20(12):e0336190. doi: 10.1371/journal.pone.0336190 (PMC12677466; doi:10.1371/journal.pone.0336190)
Supplement: S2 Table — (DOCX) [file pone.0336190.s007.docx]

**Supplementary Table 2.** Association between ecDNA, DNase, markers of NETosis and IGGGCCG risk group.

|  | **N** | **Mean** | **Median** | **SD** | **SEM** | ***p* value** |
| --- | --- | --- | --- | --- | --- | --- |
| **IGGGCCG** |  |  |  |  |  |  |
| **Plasma total ecDNA ng/mL** |  |  |  |  |  |  |
| Good | 46 | 4.744348 | 3.465 | 3.509421 | 0.941431 | **0.01164** |
| Intermediate | 8 | 8.54875 | 4.705 | 8.410532 | 2.257473 |  |
| Poor | 16 | 11.12562 | 6.64 | 10.58985 | 1.596275 |  |
| **Plasma ncDNA GE/mL** |  |  |  |  |  |  |
| Good | 40 | 4684.65 | 2766.5 | 5857.821 | 1714.863 | 0.15762 |
| Intermediate | 8 | 9045.75 | 3737.5 | 13553.26 | 3834.55 |  |
| Poor | 16 | 12371.38 | 4894.5 | 17419.06 | 2711.436 |  |
| **Plasma mtDNA GE/mL** |  |  |  |  |  |  |
| Good | 46 | 145853.5 | 107493.5 | 151470.4 | 25055.84 | 0.44048 |
| Intermediate | 9 | 252685.1 | 109750 | 244045.7 | 56645.66 |  |
| Poor | 16 | 166697.6 | 83468 | 174132 | 42484.24 |  |
| **Plasma DNase K.U./mL** |  |  |  |  |  |  |
| Good | 61 | 1.059508 | 1 | 0.359386 | 0.045524 | 0.19725 |
| Intermediate | 10 | 1.1 | 0.945 | 0.462649 | 0.112437 |  |
| Poor | 20 | 0.9105 | 0.85 | 0.276167 | 0.079505 |  |
| **Pellet total ecDNA ng/mL** |  |  |  |  |  |  |
| Good | 47 | 3.272979 | 2 | 4.355127 | 0.554317 | 0.61252 |
| Intermediate | 9 | 2.225556 | 1.45 | 1.687299 | 1.266735 |  |
| Poor | 11 | 2.458182 | 1.67 | 1.702808 | 1.145805 |  |
| **Pellet ncDNA GE/mL dich** |  |  |  |  |  |  |
| Good | 36 | 57484.39 | 3180.5 | 201874.3 | 50985.76 | 0.29957 |
| Intermediate | 7 | 12356 | 3859 | 15731.12 | 115624.8 |  |
| Poor | 13 | 232943.4 | 7403 | 542531.9 | 84845.44 |  |
| **Pellet mtDNA GE/mL** |  |  |  |  |  |  |
| Good | 47 | 449747.5 | 73419 | 927966.1 | 110852.5 | 0.45879 |
| Intermediate | 9 | 98705.78 | 66629 | 90317.67 | 253322.1 |  |
| Poor | 16 | 137916.7 | 102830 | 107712.1 | 189991.6 |  |
| **< 100 nm** |  |  |  |  |  |  |
| Good | 47 | 88587.23 | 50600 | 102967.6 | 16637.42 | 0.41053 |
| Intermediate | 9 | 106955.6 | 36200 | 142012 | 38020.13 |  |
| Poor | 16 | 140087.5 | 104100 | 128744.8 | 28515.1 |  |
| **100-500 nm** |  |  |  |  |  |  |
| Good | 47 | 823025.6 | 473000 | 976043.6 | 122154.6 | 0.18221 |
| Intermediate | 9 | 427577.8 | 318000 | 323683.1 | 279149.8 |  |
| Poor | 16 | 550512.5 | 379100 | 498706.5 | 209362.4 |  |
| **500-1000 nm** |  |  |  |  |  |  |

| Good | 47 | 1071115 | 968600 | 782873.1 | 97826.94 | 0.15327 |
| --- | --- | --- | --- | --- | --- | --- |
| Intermediate | 9 | 703844.4 | 695600 | 309318.3 | 223555.9 |  |
| Poor | 16 | 796112.5 | 807000 | 372154.8 | 167666.9 |  |
| **< 5** μ**M** |  |  |  |  |  |  |
| Good | 47 | 1188392 | 244800 | 1649807 | 223254.9 | 0.31518 |
| Intermediate | 9 | 1326467 | 328200 | 1881423 | 510186.3 |  |
| Poor | 16 | 556687.5 | 195800 | 735591.8 | 382639.7 |  |
| **> 5** μ**M** |  |  |  |  |  |  |
| Good | 47 | 472846.8 | 169200 | 666728.6 | 92123.8 | 0.3577 |
| Intermediate | 9 | 530266.7 | 102000 | 855017.1 | 210523 |  |
| Poor | 16 | 206562.5 | 86400 | 285890.3 | 157892.3 |  |
| **Small particles (< 1** μ**M)** |  |  |  |  |  |  |
| Good | 47 | 1982728 | 1604600 | 1610132 | 201145.2 | 0.18912 |
| Intermediate | 9 | 1238378 | 1261800 | 431576.9 | 459660.6 |  |
| Poor | 16 | 1486713 | 1292300 | 835208.9 | 344745.5 |  |
| **Large particles (> 1** μ**M)** |  |  |  |  |  |  |
| Good | 47 | 1661238 | 396800 | 2153986 | 297671.3 | 0.28589 |
| Intermediate | 9 | 1856733 | 388800 | 2715447 | 680243.9 |  |
| Poor | 16 | 763250 | 326600 | 998099.6 | 510182.9 |  |
| **All particles** |  |  |  |  |  |  |
| Good | 47 | 3643966 | 2507800 | 3002953 | 402512.4 | 0.18858 |
| Intermediate | 9 | 3095111 | 1931800 | 2852066 | 919828.6 |  |
| Poor | 16 | 2249963 | 1582700 | 1742200 | 689871.4 |  |
| **MPO (ng/mL)** |  |  |  |  |  |  |
| Good | 48 | 9.150833 | 6.32 | 8.227097 | 1.173754 | 0.19088 |
| Intermediate | 9 | 9.227777 | 6.92 | 5.972543 | 2.71067 |  |
| Poor | 16 | 12.47688 | 9.875 | 8.803403 | 2.033002 |  |
| **NE (ng/mL)** |  |  |  |  |  |  |
| Good | 36 | 2.822222 | 2.435 | 1.702076 | 0.392581 | 0.64297 |
| Intermediate | 6 | 2.545 | 2.34 | 1.995172 | 0.961622 |  |
| Poor | 15 | 4.207334 | 3.91 | 3.56879 | 0.608183 |  |

**Abbreviations:** ecDNA, extracellular DNA, ncDNA, nuclear DNA, mtDNA, mitochondrial DNA , MPO, myeloperoxidase, NE, neutrophil elastase, SD, standard deviation, SEM, standard error of mean
