# Supplementary material for: Low level of plasma DNase is associated with worse clinical outcome in testicular germ cell tumor patients and exogeneous DNase I improves cisplatin treatment efficacy
Source: PLoS One. 2025 Dec 4;20(12):e0336190. doi: 10.1371/journal.pone.0336190 (PMC12677466; doi:10.1371/journal.pone.0336190)
Supplement: S3 Table — (DOCX) [file pone.0336190.s008.docx]

**Supplementary Table 3.** Association between ecDNA, DNase, markers of NETosis and number of metastatic sites.

|  | **N** | **Mean** | **Median** | **SD** | **SEM** | ***p* value** |
| --- | --- | --- | --- | --- | --- | --- |
| **No. of mts** |  |  |  |  |  |  |
| **Plasma total ecDNA ng/mL** |  |  |  |  |  |  |
| 0 | 18 | 2.5 | 2.3 | 1.5 | 1.4 | **0.00004** |
| 1 to 2 | **53** | 5.4 | 4.1 | 4.9 | 0.8 |  |
| 3 and more | 24 | 10.6 | 6.9 | 9.2 | 1.2 |  |
| **Plasma ncDNA GE/mL** |  |  |  |  |  |  |
| 0 | 15 | 3151.9 | 2844.0 | 2070.1 | 2519.1 | **0.0122** |
| 1 to 2 | 49 | 5278.0 | 2805.0 | 7428.6 | 1393.8 |  |
| 3 and more | 24 | 11761.5 | 4994.5 | 15297.5 | 1991.5 |  |
| **Plasma mtDNA GE/mL** |  |  |  |  |  |  |
| 0 | 18 | 176666.1 | 76544.0 | 290192.4 | 47182.6 | 0.35594 |
| 1 to 2 | 54 | 149075.9 | 104220.5 | 151733.8 | 27240.9 |  |
| 3 and more | 25 | 208869.9 | 115206.0 | 215532.3 | 40035.8 |  |

| **Plasma DNase K.U./mL** |  |  |  |  |  |  |
| --- | --- | --- | --- | --- | --- | --- |
| 0 | 18 | 1.2 | 1.2 | 0.4 | 0.1 | **0.02139** |
| 1 to 2 | 70 | 1.0 | 1.0 | 0.4 | 0.0 |  |
| 3 and more | 29 | 0.9 | 0.9 | 0.4 | 0.1 |  |
| **Pellet total ecDNA ng/mL** |  |  |  |  |  |  |
| 0 | 18 | 2.5 | 2.0 | 2.8 | 0.8 | 0.91425 |
| 1 to 2 | 53 | 2.9 | 1.9 | 3.9 | 0.5 |  |
| 3 and more | 20 | 2.5 | 1.7 | 1.8 | 0.8 |  |
| **Pellet ncDNA GE/mL dich** |  |  |  |  |  |  |
| 0 | 11 | 79367.3 | 17729.0 | 177261.5 | 84658.3 | 0.07588 |
| 1 to 2 | 46 | 79454.4 | 2936.5 | 282547.4 | 41398.7 |  |
| 3 and more | 18 | 85576.8 | 5345.0 | 322634.6 | 66180.4 |  |
| **Pellet mtDNA GE/mL** |  |  |  |  |  |  |
| 0 | 18 | 126159.2 | 71301.5 | 178715.8 | 228908.8 | 0.43391 |
| 1 to 2 | 55 | 420725.9 | 84922.0 | 862189.3 | 130953.6 |  |
| 3 and more | 25 | 598147.8 | 75483.0 | 1427669.0 | 194235.5 |  |
| **< 100 nm** |  |  |  |  |  |  |
| 0 | 18 | 87077.8 | 36200.0 | 111764.5 | 37094.6 | 0.16831 |
| 1 to 2 | 55 | 108378.2 | 60400.0 | 113178.0 | 21221.0 |  |
| 3 and more | 25 | 122144.0 | 31200.0 | 245707.0 | 31475.8 |  |
| **100-500 nm** |  |  |  |  |  |  |
| 0 | 18 | 467566.7 | 377900.0 | 284104.2 | 208337.1 | **0.02136** |
| 1 to 2 | 55 | 822949.1 | 481400.0 | 926498.9 | 119185.0 |  |
| 3 and more | 25 | 581568.0 | 292400.0 | 1050707.0 | 176779.9 |  |
| **500-1000 nm** |  |  |  |  |  |  |
| 0 | 18 | 731644.4 | 742800.0 | 494431.7 | 141685.4 | 0.09894 |
| 1 to 2 | 55 | 1076571.0 | 953600.0 | 713316.8 | 81055.1 |  |
| 3 and more | 25 | 851424.0 | 925600.0 | 335139.7 | 120224.1 |  |
| **< 5** μ**M** |  |  |  |  |  |  |
| 0 | 18 | 377677.8 | 201800.0 | 449559.8 | 320438.3 | 0.06604 |
| 1 to 2 | 55 | 1218662.0 | 351000.0 | 1554469.0 | 183315.6 |  |
| 3 and more | 25 | 990120.0 | 249000.0 | 1317575.0 | 271900.9 |  |
| **> 5** μ**M** |  |  |  |  |  |  |
| 0 | 18 | 167477.8 | 60300.0 | 362559.5 | 163768.7 | **0.02346** |
| 1 to 2 | 55 | 537967.3 | 169200.0 | 810541.8 | 93688.4 |  |
| 3 and more | 25 | 373560.0 | 157200.0 | 582775.3 | 138962.3 |  |
| **Small particles (< 1** μ**M)** |  |  |  |  |  |  |
| 0 | 18 | 1286289.0 | 1147100.0 | 758182.4 | 322359.4 | **0.02423** |
| 1 to 2 | 55 | 2007898.0 | 1614200.0 | 1488522.0 | 184414.6 |  |
| 3 and more | 25 | 1555136.0 | 1380800.0 | 1418269.0 | 273531.0 |  |
| **Large particles (> 1** μ**M)** |  |  |  |  |  |  |
| 0 | 18 | 545155.6 | 261100.0 | 708719.6 | 448203.4 | **0.04556** |
| 1 to 2 | 55 | 1756629.0 | 582000.0 | 2158020.0 | 256407.1 |  |
| 3 and more | 25 | 1363680.0 | 503400.0 | 1865209.0 | 380313.2 |  |
| **All particles** |  |  |  |  |  |  |
| 0 | 18 | 1831445.0 | 1499800.0 | 1083122.0 | 598218.8 | **0.01569** |
| 1 to 2 | 55 | 3764527.0 | 2551600.0 | 2883630.0 | 342227.5 |  |
| 3 and more | 25 | 2918816.0 | 1931800.0 | 2440794.0 | 507605.4 |  |
| **MPO (ng/mL)** |  |  |  |  |  |  |
| 0 | 18 | 8.4 | 5.8 | 8.8 | 2.1 | 0.2832 |
| 1 to 2 | 56 | 10.0 | 7.3 | 8.3 | 1.2 |  |
| 3 and more | 25 | 12.1 | 7.9 | 10.8 | 1.8 |  |
| **NE (ng/mL)** |  |  |  |  |  |  |
| 0 | 14 | 3.1 | 2.9 | 1.7 | 0.6 | 0.65805 |
| 1 to 2 | 39 | 2.8 | 2.4 | 2.1 | 0.4 |  |
| 3 and more | 23 | 3.1 | 2.4 | 2.8 | 0.5 |  |

**Abbreviations:** ecDNA, extracellular DNA, ncDNA, nuclear DNA, mtDNA, mitochondrial DNA , MPO, myeloperoxidase, NE, neutrophil elastase, SD, standard deviation, SEM, standard error of mean
