# Supplementary material for: Low level of plasma DNase is associated with worse clinical outcome in testicular germ cell tumor patients and exogeneous DNase I improves cisplatin treatment efficacy
Source: PLoS One. 2025 Dec 4;20(12):e0336190. doi: 10.1371/journal.pone.0336190 (PMC12677466; doi:10.1371/journal.pone.0336190)
Supplement: S4 Table — (DOCX) [file pone.0336190.s009.docx]

**Supplementary Table 4.** Association between ecDNA, DNase, markers of NETosis and treatment response.

| **Response** | **N** | **Mean** | **Median** | **SD** | **SEM** | ***p* value** |
| --- | --- | --- | --- | --- | --- | --- |
|  |  | | | | | |
| **Plasma total ecDNA ng/mL** |  |  |  |  |  |  |
| Favorable | 81 | 5.0 | 3.6 | 4.7 | 0.7 | **0.00665** |
| Unfavorable | 14 | 12.7 | 10.0 | 10.8 | 1.6 |  |
| **Plasma ncDNA GE/mL** |  |  |  |  |  |  |
| Favorable | 73 | 4648.2 | 2884.0 | 5292.1 | 1072.3 | **0.00941** |
| Unfavorable | 15 | 16590.7 | 6055.0 | 19277.6 | 2365.6 |  |
| **Plasma mtDNA GE/mL** |  |  |  |  |  |  |
| Favorable | 82 | 155883.3 | 99961.0 | 186289.1 | 21879.2 | 0.25952 |
| Unfavorable | 15 | 244627.1 | 115206.0 | 256078.1 | 51155.5 |  |
| **Plasma DNase K.U./mL** |  |  |  |  |  |  |
| Favorable | 99 | 1.1 | 1.0 | 0.4 | 0.0 | **0.02682** |
| Unfavorable | 18 | 0.9 | 0.8 | 0.3 | 0.1 |  |
| **Pellet total ecDNA ng/mL** |  |  |  |  |  |  |
| Favorable | 78 | 2.7 | 1.9 | 3.5 | 0.4 | 0.60978 |
| Unfavorable | 13 | 2.8 | 1.8 | 1.9 | 0.9 |  |
| **Pellet ncDNA GE/mL dich** |  |  |  |  |  |  |
| Favorable | 63 | 94023.4 | 4136.0 | 300740.7 | 34923.3 | 0.9596 |
| Unfavorable | 12 | 12070.8 | 5597.0 | 11724.4 | 80019.3 |  |
| **Pellet mtDNA GE/mL** |  |  |  |  |  |  |
| Favorable | 83 | 316115.0 | 83753.0 | 719918.0 | 104474.7 | 0.20306 |
| Unfavorable | 15 | 941796.1 | 116814.0 | 1782281.0 | 245756.3 |  |
| **< 100 nm** |  |  |  |  |  |  |
| Favorable | 83 | 97477.1 | 49800.0 | 109010.0 | 17012.3 | 0.68581 |
| Unfavorable | 15 | 166080.0 | 30600.0 | 308412.6 | 40018.0 |  |
| **100-500 nm** |  |  |  |  |  |  |
| Favorable | 83 | 675130.1 | 448400.0 | 783444.2 | 97750.8 | 0.39338 |
| Unfavorable | 15 | 812120.0 | 318000.0 | 1357668.0 | 229939.7 |  |
| **500-1000 nm** |  |  |  |  |  |  |
| Favorable | 83 | 956537.4 | 823400.0 | 653123.1 | 67502.2 | 0.51809 |
| Unfavorable | 15 | 951600.0 | 941200.0 | 307965.9 | 158785.6 |  |
| **< 5** μ**M** |  |  |  |  |  |  |
| Favorable | 83 | 984708.4 | 249000.0 | 1437390.0 | 152349.6 | 0.28658 |
| Unfavorable | 15 | 1123120.0 | 801800.0 | 1052929.0 | 358372.6 |  |
| **> 5** μ**M** |  |  |  |  |  |  |
| Favorable | 83 | 434216.9 | 120000.0 | 743181.6 | 77455.0 | 0.29333 |
| Unfavorable | 15 | 393453.3 | 203800.0 | 423601.2 | 182197.8 |  |

| **Small particles (< 1** μ**M)** |  |  |  |  |  |  |
| --- | --- | --- | --- | --- | --- | --- |
| Favorable | 83 | 1729145.0 | 1436800.0 | 1310073.0 | 152642.6 | 0.87845 |
| Unfavorable | 15 | 1929800.0 | 1457400.0 | 1791183.0 | 359061.8 |  |
| **Large particles (> 1** μ**M)** |  |  |  |  |  |  |
| Favorable | 83 | 1418925.0 | 393000.0 | 2020749.0 | 213577.7 | 0.37187 |
| Unfavorable | 15 | 1516573.0 | 1321400.0 | 1429860.0 | 502399.7 |  |
| **All particles** |  |  |  |  |  |  |
| Favorable | 83 | 3148070.0 | 2109200.0 | 2664575.0 | 288682.4 | 0.5245 |
| Unfavorable | 15 | 3446373.0 | 2913600.0 | 2417744.0 | 679068.8 |  |
| **MPO (ng/mL)** |  |  |  |  |  |  |
| Favorable | 84 | 9.8 | 6.6 | 8.5 | 1.0 | 0.24352 |
| Unfavorable | 15 | 12.8 | 7.9 | 11.8 | 2.3 |  |
| **NE (ng/mL)** |  |  |  |  |  |  |
| Favorable | 63 | 3.0 | 2.5 | 2.1 | 0.3 | 0.24092 |
| Unfavorable | 13 | 2.6 | 2.0 | 2.7 | 0.6 |  |

**Abbreviations:** ecDNA, extracellular DNA, ncDNA, nuclear DNA, mtDNA, mitochondrial DNA , MPO, myeloperoxidase, NE, neutrophil elastase, SD, standard deviation, SEM, standard error of mean
