# Supplementary material for: Low level of plasma DNase is associated with worse clinical outcome in testicular germ cell tumor patients and exogeneous DNase I improves cisplatin treatment efficacy
Source: PLoS One. 2025 Dec 4;20(12):e0336190. doi: 10.1371/journal.pone.0336190 (PMC12677466; doi:10.1371/journal.pone.0336190)
Supplement: S5 Table — (DOCX) [file pone.0336190.s010.docx]

**Supplementary Table 5.** Association between ecDNA, DNase, markers of NETosis and specific immune cell subpopulations.

| **Variable** | **Association with % of immune cell subpopulations** | **p-value** |
| --- | --- | --- |
| **plasma total ecDNA ng/ml** |  |  |
|  | Neutrophils percentage | 0.00001 |
|  | Classical monocytes percentage | 0.00092 |
|  | Nonclassical monocytes percentage | 0.00028 |
|  | Polymorphonuclear monocytes (PNMs) percentage | 0.02572 |
|  | Basophils percentage | 0.02292 |
|  | Dendritic cells (cDCs) percentage | 0.02190 |
|  | Plasmocytoid dendritic cells (pDCs) percentage | 0.03854 |
|  | CD1c+ within DCs percentage | 0.00433 |
|  | Lymphocytes percentage | 0.00001 |
| **plasma ncDNA ge/ml** |  |  |
|  | Neutrophils percentage | 0.00720 |
|  | Classical monocytes percentage | 0.00025 |
|  | Nonclassical monocytes percentage | 0.00011 |
|  | Polymorphonuclear monocytes (PNMs) percentage | 0.03227 |
|  | Basophils percentage | 0.01418 |
|  | CD1c+ within DCs percentage | 0.01008 |
|  | Lymphocytes percentage | 0.00200 |
| **plasma mtDNA ge/ml** |  |  |
|  | No significant associations | NS |
| **plasma DNase k.u/ml** |  |  |
|  | Neutrophils percentage | 0.02830 |
|  | CD16+ HLADR+ Lin- DCs percentage | 0.04020 |
|  | CD1c+ within DCs percentage | 0.00433 |
|  | Lymphocytes percentage | 0.01791 |
| **pellet total ecdna ng/ml** |  |  |
|  | Monocytes percentage | 0.02953 |
| **pellet ncdna ge/ml** |  |  |
|  | No significant associations | NS |
| **pellet mtdna ge/ml** |  |  |
|  | No significant associations | NS |
| **small particles <100 nm** |  |  |
|  | Classical monocytes percentage | 0.01386 |
|  | Polymorphonuclear monocytes (PNMs) percentage | 0.03862 |
|  | NK cells percentage | 0.02897 |
|  | Dendritic cells (cDCs) percentage | 0.03518 |
| **small particles 100-500 nm** |  |  |
|  | Polymorphonuclear monocytes (PNMs) percentage | 0.00960 |
| **large particles 500-1000 nm** |  |  |
|  | CD4+ NKT cells percentage | 0.05117 |
|  | CD8+ NKT cells percentage | 0.02443 |
|  | B cells percentage (CD14+) | 0.04884 |
|  | T cytotoxic cells percentage | 0.02375 |
| **large particles <5 uM** |  |  |
|  | Monocytes percentage | 0.03558 |
|  | Classical monocytes percentage | 0.02076 |
|  | Eosinophils percentage | 0.00134 |
|  | Basophils percentage | 0.00499 |
|  | NKT cells percentage | 0.00049 |
|  | NK cells percentage | 0.05329 |
| **large particles >5 uM** |  |  |
|  | Polymorphonuclear monocytes (PNMs) percentage | 0.00617 |
| **small particles <1 uM** |  |  |
|  | Polymorphonuclear monocytes (PNMs) percentage | 0.00228 |
|  | B cells percentage (CD14+) | 0.04762 |
|  | Tregs percentage | 0.05497 |
| **large particles >1 uM** |  |  |
|  | Classical monocytes percentage | 0.03637 |
|  | Polymorphonuclear monocytes (PNMs) percentage | 0.05345 |
|  | Basophils percentage | 0.02054 |
|  | NKT cells percentage | 0.00042 |
| **NETs - all particles** |  |  |
|  | Monocytes percentage | 0.01245 |
|  | Classical monocytes percentage | 0.00333 |
|  | Polymorphonuclear monocytes (PNMs) percentage | 0.01345 |
|  | NKT cells percentage | 0.00044 |
| **NETs - dichMPO (ng/ml)** |  |  |
|  | Nonclassical monocytes percentage | 0.01595 |
|  | Polymorphonuclear monocytes (PNMs) percentage | 0.05318 |
| **NETs - NE (ng/ml)** |  |  |
|  | No significant associations | NS |

**Abbreviations:** ecDNA, extracellular DNA, ncDNA, nuclear DNA, mtDNA, mitochondrial DNA , MPO, myeloperoxidase, NE, neutrophil elastase, NS, non-significant,
